# Challenges in the Removal of Circular Cross-Section Lag Screw Following Compression Hip Screw Fixation: A Report of Two Cases

**DOI:** 10.7759/cureus.88764

**Published:** 2025-07-25

**Authors:** Kazuhiko Sonoda, Yusuke Kubo, Toshihiko Hara

**Affiliations:** 1 Department of Orthopaedic Surgery, Iizuka Hospital, Iizuka, JPN

**Keywords:** compression hip screws, implant complication, implant removal, lag screw, torque transmission

## Abstract

The compression hip screw (CHS) is a commonly used implant for the treatment of proximal femoral fractures. Although implant removal is not always required after fracture healing, it is occasionally performed in relatively young patients due to symptoms such as implant-related discomfort or in preparation for future procedures. While removal difficulties have been well documented for locking plates and intramedullary nails, challenges specifically involving lag screw removal from the femoral head in CHS systems are rarely reported. We report two cases of difficult lag screw removal following CHS fixation using a circular cross-section lag screw that necessitated an internal hexagonal interface for insertion and removal. In the first case, removal was unsuccessful due to strong fixation and deformation of the T-handle, resulting in retained hardware. In the second case, removal was achieved only after applying additional torque using Roman forceps. In both cases, the internal hexagonal design of the T-handle connection may have contributed to the difficulty. These cases suggest a potential limitation of CHS systems utilizing circular cross-section lag screws, particularly in younger patients with dense cancellous bone. Awareness of this complication is important when planning implant removal.

## Introduction

The compression hip screw (CHS) is a widely utilized implant for internal fixation of proximal femoral fractures, including intertrochanteric and femoral neck fractures [1,2]. Its biomechanical stability and surgical simplicity make it a reliable option in various clinical settings. Although implant removal is generally not required after bone healing, relatively young age or patient preference may lead surgeons to consider removal [3].

While removal difficulties have been widely reported with locking plates and intramedullary nails, often attributed to cold welding, bone overgrowth, or design limitations, similar reports involving CHS systems are surprisingly rare [4-6]. In particular, there is limited documentation regarding the challenges of lag screw removal from the femoral head, despite CHS being a time-tested implant.

Herein, we report two cases of difficult lag screw removal following CHS fixation in relatively young patients. Notably, in one case, removal was ultimately unsuccessful, and the lag screw had to be left in situ. In the other case, an alternative technique involving torque amplification was required. These cases emphasize the importance of understanding implant design differences and preparing for removal challenges.

## Case presentation

Case 1

**Figure 1:**
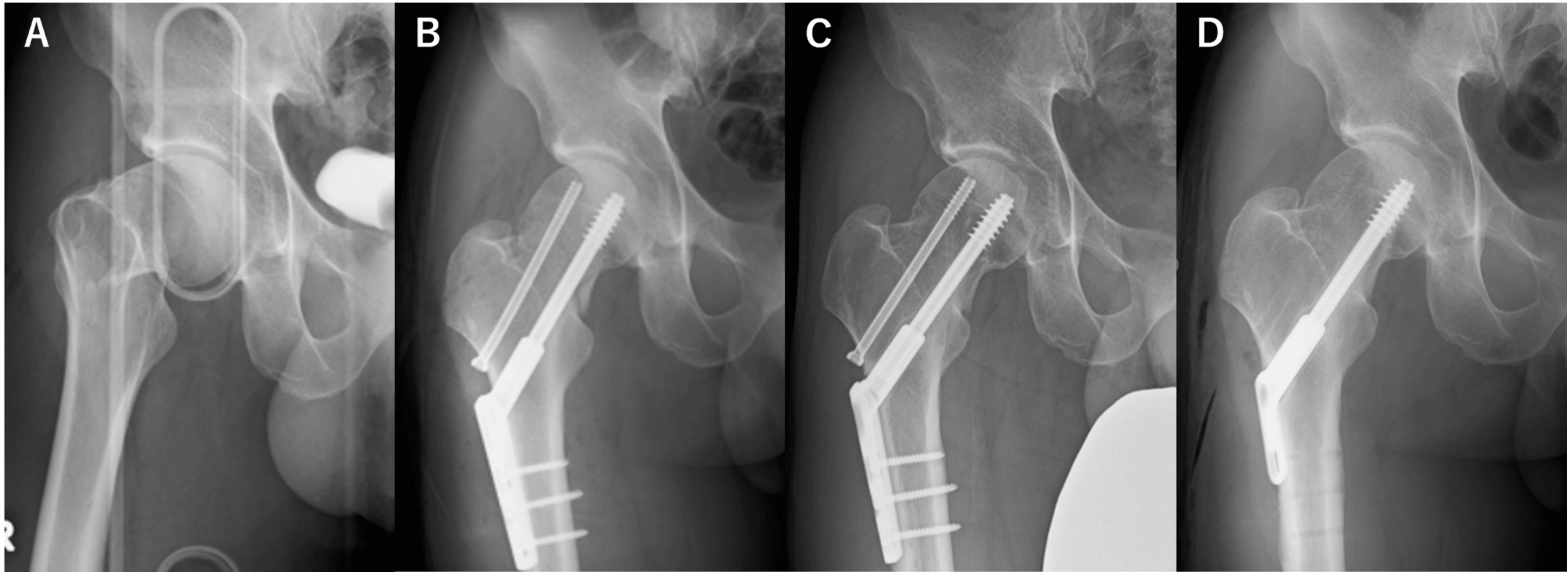
X-rays images of a 31-year-old male patient with femoral neck fracture (Garden stage IV). (A) Initial radiograph. (B) Radiograph at internal fixation with a keyless-type CHS system. (C) Radiograph at 15 months after internal fixation. (D) Radiograph after implant removal. The lag screw could not be removed due to strong fixation in the femoral head. CHS: compression hip screw

This case involved a 31-year-old male patient who presented with a displaced femoral neck fracture (Garden type IV) following high-energy trauma from a traffic accident. On the day of injury, he underwent internal fixation using a CHS system with a circular cross-section lag screw (Ti Edge, Zimmer Biomet Holdings, Inc., Warsaw, Indiana, United States), along with supplementary cannulated screws (Figure 1). The procedure was uneventful, and postoperative radiographs confirmed appropriate fracture reduction and satisfactory implant positioning. Bone union was confirmed at three months postoperatively. At 15 months, elective implant removal was scheduled at the patient's request, given his young age and desire to avoid long-term implant retention. The procedure was performed by the original surgical team after reviewing the patient's clinical course and imaging. Intraoperatively, the lag screw was found to be rigidly fixed within the femoral head. Attempts to extract the screw using the standard T-handle driver (with an internal hexagonal design) were unsuccessful due to excessive resistance. The T-handle deformed under torque, and to prevent further damage or complications, the procedure was aborted. The screw was ultimately left in situ.

Case 2

**Figure 2:**
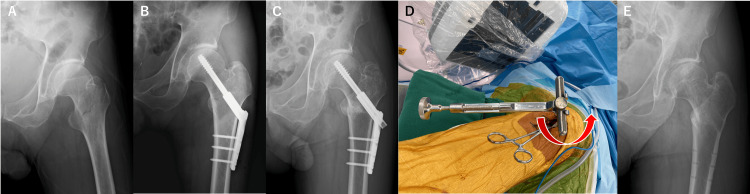
X-ray and intraoperative images of a 58-year-old male patient with an intertrochanteric fracture (AO 31A1.2). (A) Initial radiograph. (B) Radiograph at internal fixation with a keyless-type CHS system. (C) Radiograph at 18 months after internal fixation. (D) By gripping the T-handle with Roman forceps to increase torque, the screw was successfully extracted. (E) Radiograph after implant removal. CHS: compression hip screw

The second case involved a 58-year-old male patient who sustained an intertrochanteric femoral fracture (AO classification 31A1.2) following a ground-level fall. He underwent internal fixation using a CHS system (Ti Edge) on the day following the injury (Figure 2). The procedure was uneventful, and the implant was appropriately positioned, providing stable fracture fixation. At three months postoperatively, radiographs confirmed bone union. At 16 months, the patient began to experience progressive hip discomfort. Magnetic resonance imaging revealed mild degenerative changes in the hip joint, suggestive of early osteoarthritis. In anticipation of a possible future total hip arthroplasty, elective implant removal was scheduled at 18 months. The procedure was performed by the same surgical team after reviewing the patient’s prior medical records and imaging studies. During the procedure, the standard driver again failed to extract the lag screw. Recognizing the torque limitation of the internal hexagonal connection, the surgical team used Roman forceps to grip the T-handle, thereby enhancing rotational force. This maneuver enabled the successful removal of the screw. Despite the favorable outcome, the underlying structural issue remained consistent with Case 1.

## Discussion

**Figure 3:**
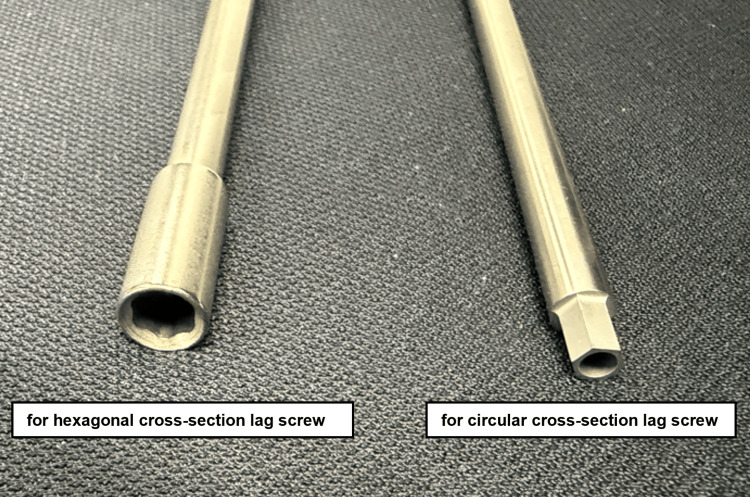
Comparison of the T-handle driver designs in CHS systems. Left: T-handle driver for a hexagonal cross-section lag screw with an external hexagonal interface. Right: T-handle driver for a circular cross-section lag screw with an internal hexagonal interface. Only the driver ends are shown; corresponding lag screw heads were not available for imaging. CHS: compression hip screw

Implant removal is sometimes considered routine; however, technical difficulties may arise depending on implant design and bone conditions. While removal challenges have been well documented with locking plates and intramedullary nails, reports related to CHS systems remain scarce [4-6]. In both of our cases, the CHS system utilized a circular cross-section lag screw that required an internal hexagonal driver interface. This configuration differs from other CHS designs, including some keyless-type systems that use a hexagonal cross-section lag screw. Compared to an external hexagonal interface, an internal hexagonal driver interface appears to have reduced mechanical strength in terms of torque transmission during screw removal, although not necessarily in fixation performance (Figure 3). While no biomechanical studies have directly compared internal versus external hex interfaces in CHS systems, this interpretation is based on clinical observations from the two cases presented. This design-related weakness becomes particularly significant in patients with dense cancellous bone, where the lag screw tends to be more firmly anchored.

In retrospect, in Case 1, the patient’s young age and presumed high bone density may have been predictive of strong screw fixation, even though no preoperative imaging showed evidence such as bone sclerosis, and removal difficulty was not anticipated. Eyres described a technique in which the plate of a CHS system can be used as a spanner to apply rotational force to the screw, aiding in extraction [6]. However, this method is inherently limited to implants with external hexagonal screw heads. With circular cross-section lag screws, such techniques are not feasible, further compounding the removal challenge. In Case 1, the T-handle deformed under excessive torque, resulting in removal failure. In Case 2, the same risk was present, but successful removal was achieved by enhancing torque through the use of Roman forceps. These cases thus illustrate a common underlying design-related issue, irrespective of the procedural outcome.

As a result of these experiences, our institution has discontinued the use of CHS systems with circular cross-section lag screws. Surgeons should be aware of this potential complication and ensure that alternative removal tools or strategies are readily available. While keyless CHS systems may offer design advantages such as a lower implant profile and simplified instrumentation, the potential risk of removal difficulty should also be considered when selecting implants. Additionally, other factors, including bone strength, implant retention duration, and surgical technique, may also influence removal difficulty and warrant further investigation.

## Conclusions

We reported two cases of difficult lag screw removal after CHS fixation, both involving a circular cross-section lag screw with an internal hexagonal interface. In one case, removal was unsuccessful due to driver deformation; in the other, successful extraction required the use of additional torque. These cases suggest that internal hexagonal designs may inherently limit torque transmission, especially in patients with dense cancellous bone. Surgeons should be aware of this potential complication when selecting implants and planning for hardware removal.
